# From Himba indigenous knowledge to engineered Fe_2_O_3_ UV-blocking green nanocosmetics

**DOI:** 10.1038/s41598-021-04663-0

**Published:** 2022-02-10

**Authors:** D. Havenga, R. Akoba, L. Menzi, S. Azizi, J. Sackey, N. Swanepoel, A. Gibaud, M. Maaza

**Affiliations:** 1 College of Graduate Studies, UNESCO-UNISA Africa Chair in Nanosciences-Nanotechnology, Muckleneuk Ridge, PO Box 392, Pretoria, South Africa; 2 Nanosciences African Network (NANOAFNET), iThemba LABS-National Research Foundation, 1 Old Faure Road, Somerset West, Western Cape 7129, PO Box 722, Johannesburg, South Africa; 3 Department of Anthropology & Archaeology, College Human Sciences University of South Africa, PO Box 392, Pretoria, South Africa; 4 Physics Dept, University of Le Mans, Le Maine, France

**Keywords:** Biophysics, Materials science, Nanoscience and technology

## Abstract

This contribution reports on the physical properties of the natural Namibian red Ochre used by the Himba Community in a form of a formulation, so called Otjize as a skin protective and beauty cream. The morphological and crystallographic studies of this red ochre validated its nano-scaled dominating phase of rhombohedral α-Fe_2_O_3_ nanocrystals with an additional hydrolized oxide component in a form of γ-FeOOH. The optical investigations showed that such a red ochre exhibits an exceptional UV filtration and a significant IR reflectivity substantiating its effectiveness as an effective UV-blocking & solar heat IR reflector in support of the low skin cancer rate within the Namibian Himba community. In addition, such nanocrystals exhibited a non-negligible antibacterial response against E. Coli & S. Aurus. This study seems confirming the effectiveness of the indigenous Otjize as an effective skin UV protection cream with a sound antimicrobial efficacy against e-Coli & S-Aurus.

## Introduction

In addition to the recently discovered Zeta-Fe_2_O_3_ structure^1^, Iron (III) oxide represents one of the most rich and multifunctional oxide families^2^. It has four standard principal polymorphs, hematite (α-Fe_2_O_3_), maghemite (γ-Fe_2_O_3_), β-Fe_2_O_3_ and ε-Fe_2_O_3_; iron (III) oxy-hydroxide also has four polymorphs known as goethite (α-FeOOH), akaganeite (β-FeOOH), lepidocrocite (γ-FeOOH) and feroxyhyte (δ-FeOOH)^2^. Of a special interest, α-Fe2O3 is a prominent n-type semiconductor with an interesting bandgap Eg = 2.1 eV, conjugated to an excellent chemical stability and low toxicity under ambient conditions. Among these oxides, α-FeOOH and α-Fe2O3 have been commonly investigated for magnetic devices, catalysts, gas sensors and photoelectrodes applications^3–10^ in addition to pigment production. However, it is worth noting that this latter pigment aspect has been identified as early as the birth of humanity.

Indeed, early human used several marine resources and pigments to express artistically on their natural surroundings & believes ranging from prehistoric, ochre-pigmented images on cave walls to paintings on canvasses in addition to their symbolic behaviors. Its usage in symbolic behavior was found to date164,000 years ago, far earlier than previously documented^11^.

More precisely, the earliest evidence of the ochre pigment’s usage by ancient humans dates to the Paleolithic, about 285,000 years ago, at a Homo erectus site called GnJh-03 in Kenya. In this location, archaeologists discovered about 70 pieces of ochre weighing about 5 kg^12^.

Early Homo-sapiens also illustrated extensively with ochre pigment. At Blombos Cave, in South Africa, archaeologists discovered an abalone shell containing finely ground ochre, charcoal as well as fat that may have made up a likely painting kit dating to about 100,000 years ago. The earliest human-made drawing is a red hashtag on small rock flake that dates to about 73,000 years ago, also at Blombos and Klein Kliphuis Caves in South Africa. In addition, about 266,000 years ago, early hominins at a site called Twin Rivers in Zambia collected a type of hematite ochre that has significantly reflective metallic flakes in it making it glittering^13–15^.

**Figure 1:**
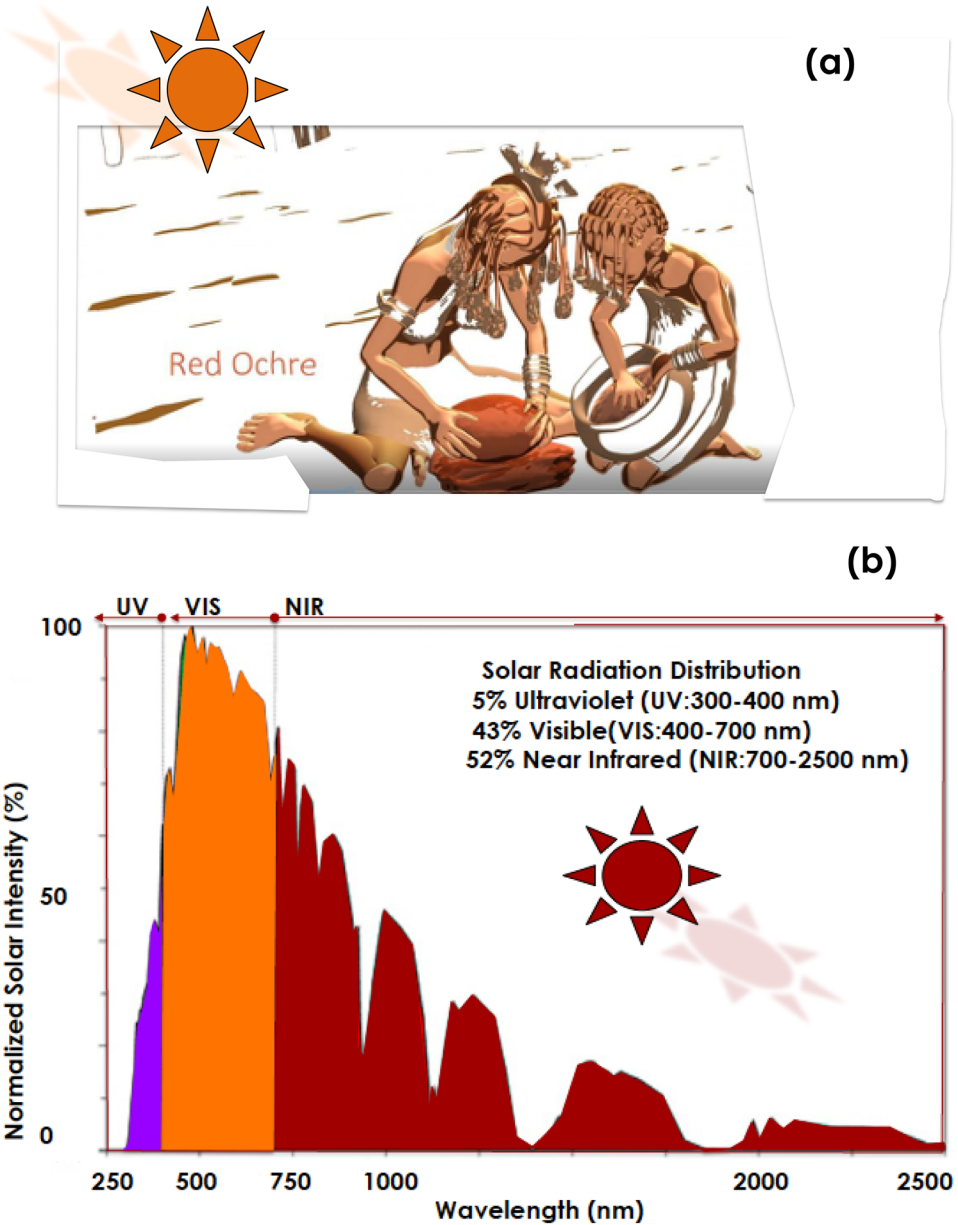
(**a**) Traditional preparation of the Red Ochre pigment by the Himba community, (**b**) Solar spectral distribution in the north of Namibia to which the Himba group community is exposed.

As highlighted previously, while Ochre was used as an effective artistic & behavioral pigment in various locations within the African continent, the Himba ethnic group specifically, has genuinely extended its usage towards cosmetic & skin-protection applications (Fig. 1a). With the Nama & the Herero communities, the Himba society is an additional indigenous social group in Namibia. Living in the northern part within the Kunene region, they are hunter-gatherers, semi-nomadics & pastoralists. Exposed to harsh atmospheric conditions in general and daily solar radiations especially where the Direct Normal irradiation is among the highest globally. In the quasi-absence of clouds, they are exposed through out the year to the standard 5% UV (300–400 nm), 43% VIS (400–700 nm) and 52% NIR-FIR (700–2500 nm) solar radiations (Fig. 1b). Accustomed to a semi-arid climate, the Himba women are famous for covering their skin & hairs with the so called Otjize paste^16^; a cosmetic mixture of goat-butterfat and ochre pigment.

Otjize cleanses the skin over long periods due to water scarcity and considered as an effective protection from the hot and dry climate, as well as from insect bites. It gives Himba people's skin and hair plaits a distinctive texture, style, and orange or red tinge, and is often perfumed with the aromatic resin of the indigenous omuzumba shrub (*Commiphora* multijuga)^17^. From anthropological viewpoint, Otjize is considered foremost a highly desirable aesthetic beauty cosmetic, symbolizing earth's rich red colour and blood, the essence of life, and is consistent with the Himba ideal of beauty (Fig. 1a).

To validate the optical filtering & selectivity as well as the health skin protection of the Ochre pigment used in the Himba Otjize, this contribution reports on its physical properties with a special focus on their optical selectivity in the UV–VIS spectral solar range and their antibacterial efficacy. If so, this would be the originality & novelty of this contribution.

## Experiments, results & discussion

For this study, typical Himba Ochre samples from the Kunene region of Namibia were collected from a Himba settlement. They were used without any further physical–chemical treatment. The samples were investigated by High Resolution Scanning Electron microscopy (HRSEM) for their morphological constituency, High Resolution Transmission Electron Microscopy (HRTEM) for the shape & atomic ordering of the elementary crystals if any, Energy Dispersive Spectrometry (EDS) for elemental analysis in addition to room temperature X-Rays Diffraction (XRD) for their crystallographic structure.

These studies were complemented with a full range of optical investigations (Photoluminescence, Infrared & UV–VIS spectroscopies). The antibacterial activity was carried out on 2 standard bacterial species: E-coli & Staphylococus Aurus.

### Morphology & crystallographic investigations

**Figure 2:**
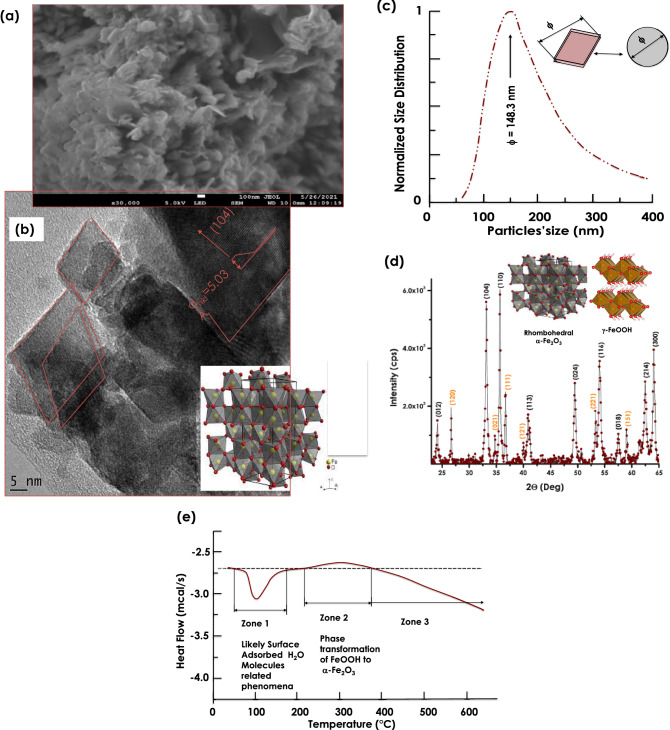
(**a**) High Resolution SEM image of the Red Ochre pigment, (**b**) its High Resolution TEM observation of the crystalline rhombohedral nanoplatelets like-particles, (**c**) their Size distribution, (**d**) its corresponding room temperature X-Ray diffraction pattern, and (**e**) their differential scanning calorimetry within the temperature range of 25–600 °C.

Figure 2a reports the HRSEM of the Himba ochre. As one could notice, it consists of agglomerated lamellar nanoplatelets. Their longitudinal & basal dimensions are relatively wide spread in terms of dimension. The average basal dimension is estimated within the range of 17–31 nm while the longitudinal average dimension is above 100 nm. Figure 2b reports an HRTEM of the Himba Ochre after sonication. The lamellar nano-platelets seem consisting of nanoscale relatively crystalline grains with preferred crystalline orientation and a platelets type morphology. The major observed atomic ordering correponds to an inter-reticular distance of 5.03 Å in agreement with the (104) atomic orientation of pure single phase hematite α-Fe_2_O_3_. Figure 2c displays the size distribution of the nanoparticles obtained by standard light scattering. The corresponding profile indicates a relatively polydisperse nanosystem with an average size of <ϕ>  = 148.3 nm. In this analysis, the nano-platelets were approximated as spheroids with a diameter ϕ. This latter parameter could be almost be the equivalent of the size of the nanoplatelets.

Figure 2d reports the XRD diffraction pattern of the Ochre sample within the angular range 2θ of 22.5–65 Deg. One can distinguish 2families of diffraction Bragg peaks. The first one is identified & indexed as (012), (104), (110), (113), (024), (116), (018), (214) and (030) of the rhombohedral single phase α -hematite α -Fe_2_O_3_. The second family set is characterized by the following diffraction Brag peak. Namely (120), (021), (111), (121), (221), (151). This later set corresponds to γ-FeOOH. It should be noted that (104) diffraction Bragg peak is one of the most intense peak, in agreement with the atomic ordering of the nanocrystals with a preferred lattice atomic ordering of d_hkl_ = 5.04 Å observed in the previous HRTEM studies of Fig. 2b. In a pre-conclusion, the Himba Ochre sample consists of hematite α-Fe_2_O_3_ mainly with an amount of a hydrolized form in a form of γ-FeOOH. In view of investigating the thermal stability of such a nano-system, Differential Scanning Calorimetry (DSC) studies under standard air were conducted within the thermal range of 25–650 °C. Figure 2e reports the corresponding DSC profile. This latter exhibits 3 major zones with an exothermic and an endothermic peaks with the first zones. Zone 1 is likely to be related to the melting/evaporation of H_2_O molecules adsorbed onto the sample surface. Zone 2 corresponds likely to a phase transformation of FeOOH to α -Fe_2_O_3_^18–24^.

### Elemental investigations

**Figure 3:**
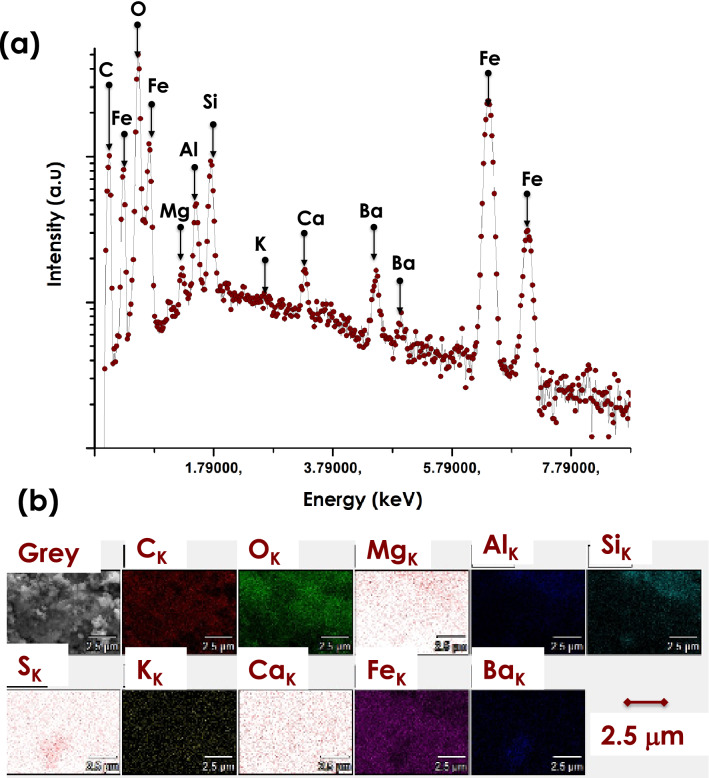
(**a**) EDS elemental spectrum of the Red Ochre pigment & (**b**) its corresponding 2-D disctribution scan of the various detected elements.

Figure 3a depicts their corresponding elemental EDS spectrum. There are 4 major Fe peaks located at various channels with the O peak as the most intense. The C peak originates from the Carbon coating used to avoid surface charging effect during the HRSEM investigations. Likewise, one can distinguish the presence of several contaminants including Mg, Al, Si, K, Ca & Ba. Figure 3b reports their corresponding 2D scans showing that they are relatively homogeneously distributed within the Ochre sample yet at low concentrations. Their qualitatively values are significantly at low levels i.e. impurities level relatively to Fe & O. The relative atomic ratio of O/Fe is about 1.62 suggesting that the the Himba Ochre is likely hematite Fe_2_O_3_ (O/Fe = 1.5) rather than magnetite Fe_3_O_4_(O/Fe = 1.3).

### Vibrational spectroscopy investigations

**Figure 4:**
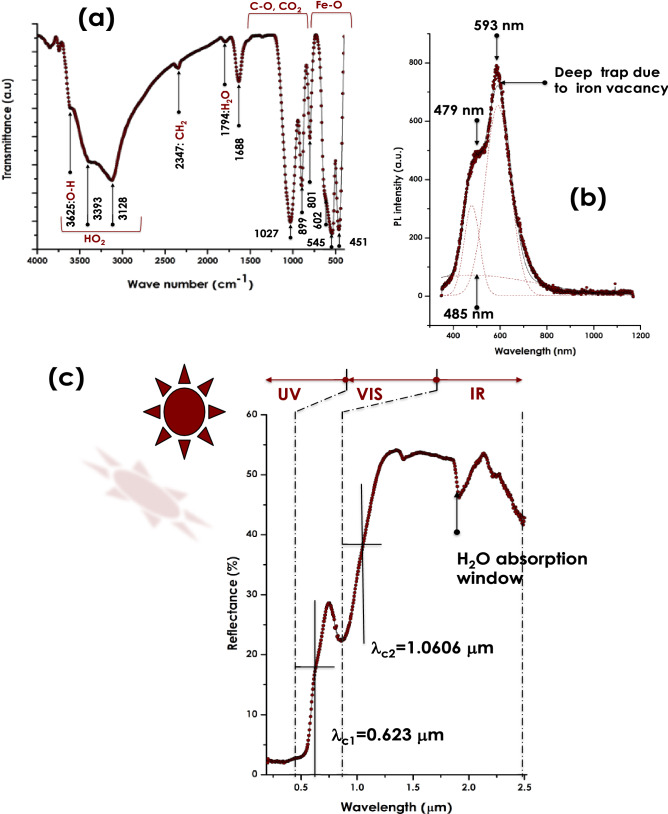
(**a**) ATR-FTIR vibrational modes of the Red Ochre pigment, (**b**) its Photoluminescence emission spectrum under an excitation wavelength λ_exc_ = 270 nm, and (**c**) its UV–VIS-NIR reflectance in the spectral range of 190–2500 nm.

Figure 4a reports the Attenuated Total Reflection (ATR)-Fourier Transform InfraRed (FTIR) spectrum of the Himba Ochre within the 400–4000 cm^-1^ spectral range. One can notice the relatively sharp mode at 451 cm^-1^ & the convoluted peaks at 545 & 602 cm^-1^ which are characteristic of the Fe–O vibrational modes. The various absorption modes from 801–1688 cm^-1^ are assigned to C=O, C–O–C stretching modes related to adsorbed CO_2_ molecules on the surface of the α-Fe_2_O_3_ crystals. In addition to the 2 low intensity vibrational modes of 1794 & 2347 cm^−1^ assigned to H_2_O & CH_2_ respectively, there is a broad band stretching from 2750–3700 cm^−1^ which may rise from various vibrational modes of H_2_O molecules. The latter molecules are likely due to atmospheric water molecules adsorbed on the surface of the α-Fe_2_O_3_ nano-crystals or those of the γ-FeOOH.

### Defects & luminescence investigations

From luminescence viewpoint in defects free α -Fe_2_O_3_, luminescence emissions would originates from the valence band of the Fe (3d) and O (2p) states, to the conduction band dominated by Fe (4 s) states. Because the local configuration of the d-band, α-Fe_2_O_3_ does not exhibit photoluminescence emission in its bulk form^25^. Figure 4b reports the luminescence spectrum at room temperature of the Otjize ochre sample under an excitation of 270 nm. It shows a relatively intense emission with a shoulder. More precisely, The simulation of the emission profile consists of 1 broad & 2 sharp emissions centered at 485, 479 and 593 nm respectively. The origin of these luminescence emissions could be due to two major factors: (i)the Oxygen surface atomic coordination as per the Pauling & Hendrick’s model for α -Fe_2_O_3_^26^ (ii) An increase in the F-O bonding separation inducing an enhancement in the magnetic coupling between Fe^3+^ ions neighbours as reported by Han et al.^27^ and Blake et al.^28^,, (iii)surface defects which may arise from deep traps caused by Fe vacancies^28^.

### Optical investigations & UV selectivity

In terms of optical absorbance, standard hematite α-Fe_2_O_3_ exhibits several absorbance bands centered at about 347 nm, 543 nm assigned to metal charge transfer direct transitions and to the double excitation processes ^6^A1(6 s) + ^6^A_1_(6 s) to ^4^T_1_(^4^G) + ^4^T_1_(^4^G) respectively^29^. Those in the range of 478–550 nm are caused by ^6^A_1_(6 s) to ^4^E, ^4^A_1_(^4^G) ligand field transitions^30^. In addition to these various absorbance bands, there is a strong absorbance at 543 nm which at the origin of the red color of hematite. Figure 4c reports the Reflectance of the Otjize ochre sample in the spectral range of 190–2500 nm. As one can notice, there is a sharp spectral cut-off at about 570 nm. The reflectance is minimal below such a cut-off; < 3%. Because of the opacity of the sample, the spectral range in the UV & part of the VIS is fully absorbed by the Otjize ochre sample in the measured spectral range of 190–570 nm. Hence, it can be concluded that the Otjize ochre sample is an exceptional UVA & UVB selective filter & hence an effective shield against solar UV radiations induced skin cancer. Following a dip in the reflectance centered at 855 nm, there is a plateau-like with an average of 53.6% suggesting that the Otjize ochre coating minimizes the skin overheating by reflecting-back at least half of the solar heat in the IR region. While the dip centered at 855 nm is due to of ^6^A_1_(6 s) to ^4^T_1_(^4^G) ligand field transition^31^, the one at about 1950 nm corresponds to a H_2_O absorption window. This latter absorption is likely to be caused by atmospheric H_2_O molecules adsorbed on the α-Fe_2_O_3_ Otjize ochre sample. One should mention that this optical cut-off behavior of hematite which makes it of a special interest in solar water photo-splitting applications^7,8,32–34^.

### Antibacterial studies

**Figure 5:**
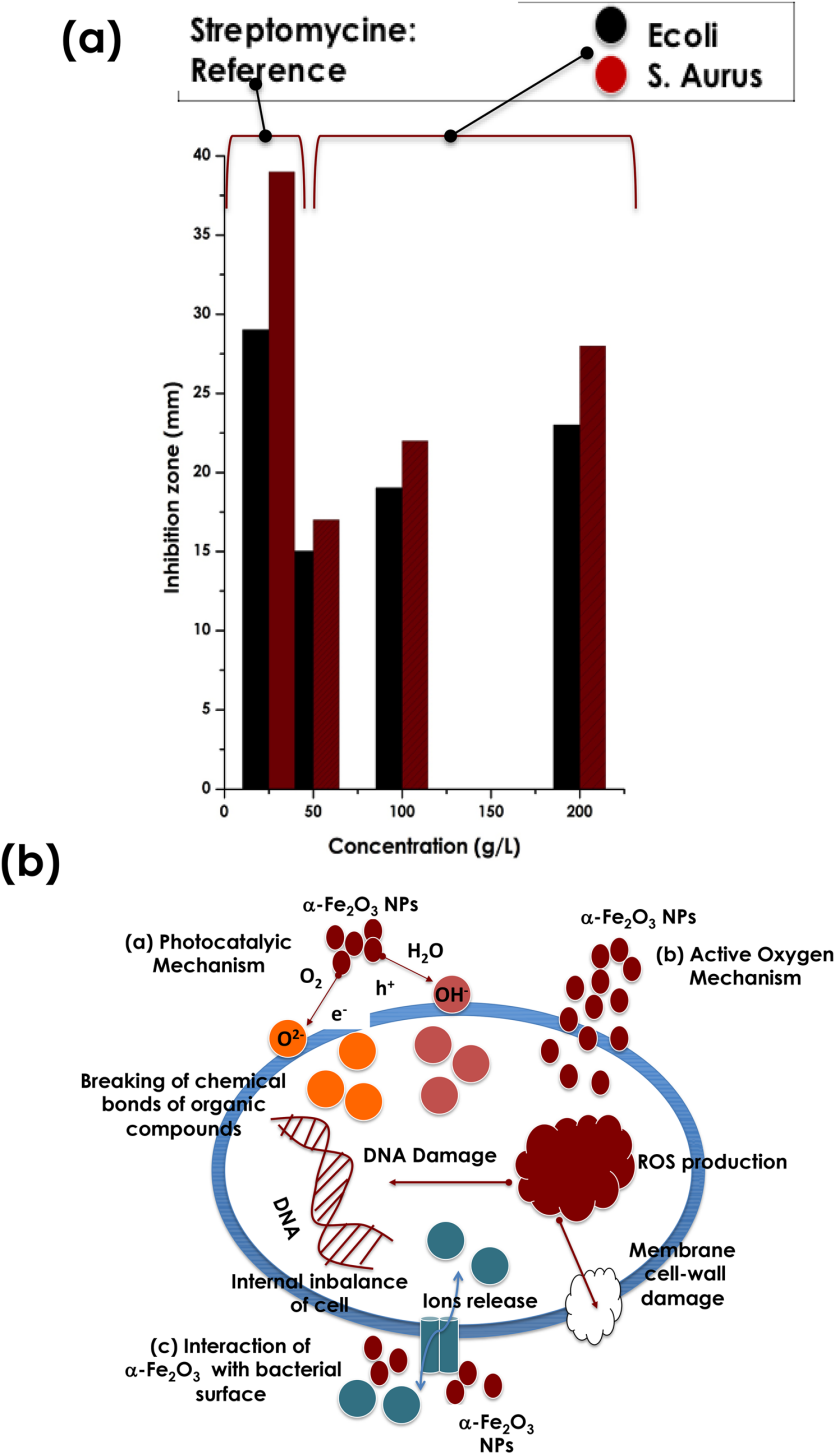
(**a**) Antibacterial response of the Red Ochre pigment against the E-coli & S-Aurus compared to standard reference Streptomycine (**b**), Schematic representation of the 3 potential mechanisms inducing the antibacterial activity of the Ochre.

Relatively to the standard nano-TiO_2_ & nano ZnO possessing cut-off wavelengths at the UV-Bleu spectral range at the vicinity of 380 & 390 nm respectively, the one of the current Himba Otjize red Ochre is by contrast in the visible range at the vicinity of 570 nm as per (Fig. 4c). Consequentially, the nano-scaled α -Fe_2_O_3_ of the Himba Otjize red Ochre should exhibit an antibacterial response. Indeed, as reported in Fig. 5a, the antibacterial activity against E-coli & staphylococcus-aurus. More precisely, it reports the inhibition zone against E-coli & staphylococcus-aurus at various Himba Otjize red Ochre α-Fe_2_O_3_ concentrations;50, 100 & 200 μg/μl. One can notice that there is a crystal clear an antibacterial against both species yet relatively low compared to the standard Streptomycine. However, the efficacy of the Himba Otjize red Ochre α-Fe_2_O_3_ nanoparticles become effective at higher concentrations. This trend is comparable to those obtained by Rufus et al.^35^ on pure hematite bio-synthesized using natural extract of Psidium guajava’s leaves as an effective chelating agent.

In terms of the antibacterial activity of the nano-scaled α-Fe_2_O_3_ of the Himba Otjize red Ochre, one can distinguish 3 potentials mechanisms which are schematically represented in Fig. 5b^36–43^;

(i) Photocatalytic mechanism, (ii)Reactive Oxygen Species mechanism & or (iii)Cell surface interaction with the α-Fe_2_O_3_ nanoparticles mechanism. In the first mechanism, once the the α-Fe_2_O_3_ nanoparticles are excited with a light energy larger than the bandgap, an e^_^−H^+^ excitonic pair is created. This e^_^–H^+^ pair reacts with O_2_, hydroxyl groups as well as the adsorbed H_2_O molecules to produce sturdy oxidative compounds such as OH^_^, O^2_^ and H_2_O_2_. The H^+^ and OH^_^ are characterized by strong oxidative properties, able to break the chemical bonds of various compounds inducing the degradation of the micro-organisms and therefore antibacterial effectiveness. The second potential mechanism involves Reactive oxygen species. Once diffused in the cell, the nanoscaled α-Fe_2_O_3_ nanoparticles produce ROS species inducing a severe damage to bacterial membranes and hence bacteriolysis & aggregation of α-Fe_2_O_3_ nanoparticles within the bacteria leading their death. In the third mechanism, and because of the high surface to volume ratio of the α-Fe_2_O_3_ nanoparticles, their interaction with the cell surface leads to a significant bacterial membrane damage and the release of ion channels, resulting in internal ionic imbalance of the cells, and eventually death^43,44^.

As a major pre-conclusion of this study, it is worth pointing that the effective UV filtration of the red ochre used by the Himba Women could explain the low skin cancer within such a community in Namibia^45^.

## Conclusion

This study was geared towards the investigation of the bio-physical properties of Red Ochre pigment used by the Himba in their Otjize formulation to protect their skin from solar radiations. It was found that the Red Ochre pigment consisted of nanoscaled particles of mainly α-Fe_2_O_3_ with γ-FeOOH nanoparticles. Such a Red Ochre pigment exhibited a significant UVA & UVB blocking optical properties hence is an effective skin cancer protection. In addition, it exhibits an effective reflectivity in the IR region hence minimizing solar heat burning of the Himba bodies in the harsh Namibian desertic climate. In addition, it was found that such a red Ochre pigment exhibit additional antibacterial efficacy against E-coli & Staphylococcus Aurus. As a major conclusion of this study, it is worth pointing that the effective UV filtration of the red ochre used by the Himba Women could explain the low skin cancer within such a community in Namibia.
